# Family Presence During Invasive Procedures or Resuscitation, Nurse Perception and Preparedness Questionnaire (FPDIP/R‐NPPQ): A Validity and Reliability Study

**DOI:** 10.1111/nicc.70470

**Published:** 2026-04-06

**Authors:** Davide Bartoli, Francesca Trotta, Mariachiara Figura, Marco Di Muzio, Daniele Napolitano, Marco Fatata, Gianluca Pucciarelli, Francesco Petrosino

**Affiliations:** ^1^ Department of Well‐Being, Health and Environmental Sustainability (BeSSA Department) Sapienza University Rome Italy; ^2^ Unit of Anesthesia, Intensive Care and Pain Medicine Sant'Andrea University Hospital Rome Italy; ^3^ Department of Health Promotion, Maternal and Infant Care Internal Medicine and Medical Specialties (PROMISE), University of Palermo Palermo Italy; ^4^ Department of Biomedicine and Prevention University of Rome Tor Vergata Rome Italy; ^5^ Department of Medicine and Psychology Sapienza University of Rome Rome Italy; ^6^ Department of Health Management Local Health Authority of Salerno Salerno Italy

**Keywords:** family presence during resuscitation, nurse, perception, preparedness, reliability, validity

## Abstract

**Background:**

Family presence during invasive procedures and resuscitation (FPDIP/R; FPDR) is increasingly advocated, yet no validated instrument assesses nurses' perceptions and preparedness including organisational readiness for implementation.

**Aim:**

To develop and psychometrically validate the FPDIP/R‐NPPQ.

**Study Design:**

A multicentre cross‐sectional survey was conducted in three Italian hospitals (April–July 2024). Registered nurses (*N* = 212) completed a study‐specific instrument initially comprising 52 items; psychometric analyses were performed on 37 Likert‐type items. We applied a two‐step strategy: exploratory factor analysis (EFA; parallel analysis, principal axis factoring, oblimin rotation, marker‐index screening) followed by confirmatory factor analysis (CFA) using robust maximum likelihood for ordinal/non‐normal data. Reliability was estimated via McDonald's *ω*. A grant from the Center of Excellence for Nursing Scholarship, Rome, March 2025, is funding this research.

**Results:**

EFA supported a 19‐item, three‐factor solution explaining 57.47% of variance: (1) emotional value and relational benefits of family presence (5 items); (2) communication skills and structured management (6 items); and (3) operational support through a dedicated staff member (8 items). CFA confirmed good fit (RMSEA = 0.05, 90% CI 0.04–0.06; CFit = 0.36; CFI = 0.93; TLI = 0.92; SRMR = 0.07), with standardised loadings ranging 0.24–0.92 and *ω* = 0.82–0.87 across factors. Model refinement improved fit without permitting cross‐loadings, preserving construct distinctiveness while accounting for localised method effects.

**Conclusions:**

The FPDIP/R‐NPPQ is a valid and reliable 19‐item, three‐factor instrument measuring nurses' readiness for family presence during invasive procedures and resuscitation. By distinguishing attitudes towards family presence, communication/management preparedness and organisational readiness (including the facilitator role), the tool enables services to identify implementation barriers and to plan targeted training and governance strategies prior to adopting FPDR/FPDIP policies.

**Relevance to Clinical Practice:**

The FPDIP/R‐NPPQ supports clinical governance by providing actionable subscale profiles to guide implementation of FPDIP/R. It can be used to baseline readiness across units, identify specific training and protocol needs and inform organisational decisions such as the allocation of a trained family facilitator, thereby supporting safer and more standardised family presence practices during critical procedures.

## Impact Statements

- What is known about the topic
  ○ Family presence during invasive procedures and resuscitation (FPDIP/R) is increasingly supported by international guidelines but remains inconsistently implemented across clinical settings.
  ○ Existing instruments assess perceived risks, benefits or self‐confidence towards FPDIP/R but do not capture nurses' preparedness and organisational dimensions in an integrated way.
  ○ Cultural and institutional barriers still limit nurses' readiness to involve families during resuscitation or invasive procedures.
- What this paper adds
  ○ The FPDIP/R‐NPPQ is a psychometrically robust, three‐factor instrument that operationalises nurses' readiness for family presence during invasive procedures and resuscitation beyond risk–benefit or self‐confidence measures.
  ○ It introduces a distinct organisational readiness domain—operational support through a dedicated staff member—which captures the presence and perceived need for structured roles (e.g., a family facilitator) and institutional support for safe implementation.
  ○ The instrument provides a practical framework for clinical managers and educators to translate scores into action: identifying unit‐specific barriers, tailoring communication and simulation training, prioritising protocol development and monitoring implementation over time.

## 1 Introduction

Sudden cardiac arrest (SCA) is one of the biggest public health problems in the worldwide [1].

Bereaved family members following sudden cardiac death (SCD) often experience social isolation, worsening physical, psychological and quality‐of‐life status and consequently increasing health service utilisation and mortality rates for surviving family members [2, 3].

*Family presence during resuscitation* (FPDR) offers relatives an opportunity for a final farewell and may help them comprehend the gravity of the situation [4]. The incidence of depressive symptoms among family members who were unable to be present during a terminal event, even 1 year later, exceeds 40% compared to those who were able to be present [5]. Rates of anxiety, stress and post‐traumatic stress disorder (PTSD) among family members vary by cultural and social context [6]. In countries such as the United States and the United Kingdom, where it is more common for family members to be present during cardiopulmonary resuscitation (CPR), the literature generally reports more favourable attitudes and relatively lower post‐event anxiety than in settings where FPDR is uncommon [7].

The term *Family Witnessed Resuscitation* (FWR) refers to the ethical principle of allowing family members to witness CPR or other life‐saving emergency interventions, such as defibrillation or the administration of life‐saving drugs [8]. FPDR is a broader concept that can include both passive presence and more active forms of involvement of family members during a resuscitation procedure [9]. Beyond visual observation, FPDR may include interaction among family, patient and clinical team—such as listening to relatives' perspectives during resuscitation [10]. It also covers circumstances in which family members provide emotional comfort—particularly when the patient is conscious or semi‐conscious—or contribute to care decisions (e.g., consent for invasive treatments) [10]. Family Presence During Invasive Procedures (FPDIP) is a complex and still under‐debated topic [11]. Nevertheless, recent studies report multiple benefits of FPDIP, including better clinician–patient communication and enhanced psychological support for relatives [11, 12]. The presence of a family member can help patients feel less alone and reduce anxiety and stress during invasive and/or painful procedures [12, 13].

Waldemar et al. argue that family presence during CPR affects the patient, the family and healthcare providers within a triadic relationship in which each influences the others [14]. In an analysis of 17 adult FPDR cases, it was found that the involvement of a pathway/family facilitator benefited both relatives and staff, highlighting the central role of organisational culture in determining staff acceptance of FPDR [15]. Recent studies indicate that FPDR is still not routine in many healthcare settings, contributing to staff resistance due to fears of complications or limited institutional support [16, 17]. Although many nurses and physicians acknowledge FPDR's potential benefits, they often report feeling insufficiently prepared to manage relatives' emotional reactions or to communicate clinical dynamics clearly and sensitively [18]. Continuing education may be pivotal in overcoming these barriers. In a recent systematic review, simulation‐based training that incorporated the presence of family members during resuscitation improved practitioners' confidence and their ability to manage such situations effectively [19].

The literature thus underscores the importance of ongoing education for healthcare professionals regarding FPDIP/R, alongside policies that promote clinician comfort and ensure family safety [10]. Although prior instruments have examined nurses' intentions towards FPDR [20] and perceived risk–benefit profiles related to FPDR [21], to our knowledge no validated questionnaire has specifically assessed nurses' perceptions and preparedness for FPDR, including an organisational dimension. In fact, Twibell et al. state that while the physician is only concerned with resuscitating the patient, during the invasive procedure, the nurse is also responsible for managing family members, the patient and the resuscitation team [22]. For this reason, it is necessary to investigate nurses' perceptions and preparedness for FPDIP/R in relation to an organisational aspect, which remains unexplored to date as it has only been investigated in terms of perceptions of the risks/benefits of family members being present during the procedure.

Developing a structured assessment tool is therefore essential to support targeted, context‐sensitive education for nurses, who play a central role as both clinical professionals and mediators for family members during FPDIP/R. Evidence suggests that healthcare professionals' personal attitudes significantly influence satisfaction with FPDIP/R experiences [23], and these attitudes are in turn shaped by professional preparation, cultural and ethical beliefs and organisational context [24, 25, 26]. However, these determinants are rarely assessed simultaneously within a single, validated instrument. In the absence of formal institutional policies, decision‐making around FPDIP/R is often driven by pre‐existing values, expectations and relational dynamics within the nurse–patient–family triad [27] increasing variability in practice and potentially exposing nurses and families to avoidable emotional and organisational strain. A clear understanding of nurses' perceptions, preparedness and organisational support is therefore a prerequisite for designing tailored educational interventions and for strengthening nurses' clinical decision‐making during FPDIP/R. When nurses are adequately trained and supported within their organisational context, they are better positioned to manage family presence safely, enhance interprofessional collaboration and foster trust‐based relationships with families grounded in informed, empathetic and concrete support [24]. Identifying these dimensions through a validated questionnaire addresses a critical gap in the literature and responds to the need for targeted—not standardised—training strategies to support ethical, effective and sustainable implementation of FPDIP/R in clinical practice. This study aims to validate and establish the reliability of a questionnaire assessing Italian nurses' perceptions and preparedness for FPDIP/R.

## 2 Design and Methods

We conducted a multicentre, descriptive cross‐sectional study using an online survey. Reporting followed the Guidelines for Reporting Reliability and Agreement Studies (GRRAS) for validation and reliability studies [28](see Appendix S1).

### 2.1 Setting and Sample

Between April and July 2024, a convenience‐sampled, multicentre survey was conducted at Belcolle Hospital (Viterbo), San Filippo Neri Hospital (Rome) and St. Andrea University Hospital (Rome). The sampling frame comprised five intensive care/emergency services at the first St. Andrea University Hospital (224 nurses), three at the second San Filippo Neri Hospital (93 nurses) and three at Belcolle Hospital (104 nurses), for a total of 421 eligible nurses. Territorial Emergency Services refers to hospital‐affiliated prehospital emergency teams (e.g., EMS/prehospital critical care units based within or coordinated by the participating hospitals, including, where applicable, helicopter emergency medical services) whose nurses were included in the hospitals' staffing lists and routinely manage resuscitative and invasive emergency care. Inclusion criteria were current contractual employment at a participating hospital, registered nurse with a bachelor's degree or equivalent practising in Italy, ICU/ED appointment or hospital‐affiliated prehospital emergency service appointment, fluency in Italian, certified e‐mail account, basic computer literacy and internet access and voluntary participation. Because the entire target population was accessible, no a priori sample‐size calculation for hypothesis testing was performed. However, the final number of responses—although lower than the total eligible population—exceeded the minimum sample size of 202 required to ensure adequate precision for descriptive and psychometric analyses (95% confidence level, 5% margin of error, *p* = 0.50), estimated using GPower*.

### 2.2 Instrument

Data were collected using a study‐specific questionnaire developed by three researchers (D.B., M.FI. and F.T.) with subject‐matter and methodological expertise. Instrument development proceeded in two steps.

Step 1. Domains were identified through a comprehensive literature review focussing on three thematic areas: (1) the Family Centred Care (FCC), FPDR and FPDI model [29, 30, 31, 32, 33, 34]; (2) nurses' opinions, practices, training and experiences [30, 32, 33, 35, 36, 37, 38]; and (3) family experience [16, 32, 33, 39]. From these themes, three dimensions were specified for the questionnaire: (1) nursing experiences and opinions; (2) training and support needs for healthcare providers; and (3) the need for a practitioner who can welcome, support and accompany family members during invasive or resuscitative procedures, consistent with FCC principles. During iterative team discussions, items were added, removed or rewritten; the themes ‘family mediator’ and ‘adherence to FCC principles’ were integrated into a single third dimension.

Step 2. Content validity was appraised using the think‐aloud method, previously applied in nursing instrument development [40, 41]. Expert participants read items aloud during interviews, explained their interpretations and response strategies and provided feedback on wording and clarity; the interviewer probed ambiguities and proposed reformulations as needed.

The final questionnaire comprised 52 items across three dimensions: Dimension 1 (27 items) captured nurses' lived experiences and opinions regarding CPR with family present, identifying barriers, strengths, beliefs and resistance to FPDR from an FCC perspective; Dimension 2 (13 items) addressed training and support needs (including protocols/guidelines) for FPDIP/R implementation; Dimension 3 (11 items) assessed the perceived need for a practitioner to welcome, support and accompany family during invasive procedures, including physical, psychological and communication support consistent with FCC. All items were positively worded on a 5‐point Likert questionnaire (agreement: 0 = ‘Very much agree’ to 4 = ‘Very much disagree’; intensity: 0 = ‘Very much’ to 4 = ‘Not at all’). Additional dichotomous items (0 = yes; 1 = no) probed opinions/knowledge, and two open‐ended items (in Dimension 2) elicited qualitative insights on training. A 9‐item sociodemographic section preceded the questionnaire (sex, age, years of experience, citizenship, master's degree and specialisation, geographic work area, current department). A pilot test (*n* = 30) was conducted by the research team (D.B., M.FI. and F.T.) to assess comprehensibility, clarity and thematic relevance of the items. The pilot was administered exclusively in an online format using the same REDCap platform planned for the main study and was conducted within the participating hospitals. Participants completed the questionnaire using the think‐aloud approach and were subsequently asked to provide structured feedback on item wording, response options and overall coherence. Minor linguistic refinements were made based on this feedback. Pilot data were excluded from the main analyses. Average completion time was 13–14 min, with mandatory responses for all items.

### 2.3 Data Collection

Unit supervisors and nursing management were contacted to facilitate access to institutional e‐mail lists for the administration of an online‐only questionnaire. Between April and July 2024, potential participants received an e‐mail describing the study aims, a link to the questionnaire hosted on REDCap [42] and an electronic consent form to be completed prior to participation. The survey window was 3 weeks; non‐responders received reminders. Contact details for the project leads (D.B. and F.P.) were provided for queries. Two technical issues affecting survey progress were identified and resolved with support.

### 2.4 Data Analysis

Analyses were conducted in IBM SPSS Statistics 27.0 [43] and Mplus 7.1 [44]. Descriptive statistics (frequencies, percentages, means, SDs) summarised sociodemographics. Multivariate normality was assessed using Mardia's skewness and kurtosis tests. The 37‐item FPDIP/R‐NPPQ was evaluated via a two‐step exploratory factor analysis (EFA) and confirmatory factor analysis (CFA). For EFA, parallel analysis (100 Monte Carlo simulations) determined factor retention (eigenvalues > 95th percentile) [45] followed by principal axis factoring with rotation selected according to inter‐factor correlations; items were retained for primary loadings ≥ 0.40 with minimal cross‐loadings [46], and cross‐loadings were further screened using the marker index [47]. Items failing cut‐offs were excluded. Internal consistency was estimated with Cronbach's *α*, adopting *α* ≥ 0.70 as acceptable [48].

Before CFA, a null (independence) model was estimated to verify the informativeness of incremental fit indices. CFA then used a robust estimator for ordinal/non‐normal data [49, 50]. Model fit was judged by RMSEA < 0.08, SRMR < 0.08, RMSEA‐CFit *p* > 0.05 and CFI/TLI > 0.95; residual covariances between adjacent items were freed to address proximity/method effects [51]. Internal consistency was additionally supported by McDonald's *ω*, with *ω* ≥ 0.70 considered acceptable [52, 53].

### 2.5 Scoring and Interpretation of the FPDIP/R‐NPPQ

The FPDIP/R‐NPPQ is a multidimensional instrument designed to assess nurses' perceptions and preparedness for family presence during invasive procedures and resuscitation at the group and organisational level. All retained items are rated on a 5‐point Likert scale (0–4), with lower scores indicating more favourable perceptions and higher perceived preparedness.

Based on exploratory and confirmatory factor analyses, three distinct subscale scores are calculated by summing item responses within each latent dimension: (1) emotional value and relational benefits of family presence (5 items; score range 0–20), (2) communication skills and structured management of family presence (6 items; score range 0–24) and (3) operational support through a dedicated staff member (8 items; score range 0–32). No overall total score is computed, as the factors represent related but conceptually distinct domains.

The instrument is not intended for diagnostic purposes and no a priori clinical cut‐off values were defined. Scores are interpreted descriptively, with lower subscale scores reflecting stronger endorsement of family presence, higher perceived communication preparedness and greater perceived organisational readiness. The FPDIP/R‐NPPQ is intended to support group‐level comparisons (e.g., across clinical settings or before and after educational interventions), identification of training needs and evaluation of organisational readiness for implementing family‐centred practices, rather than individual clinical decision‐making.

### 2.6 Ethical and Institutional Approvals

The study protocol received approval by the local Ethics Committee ‘Campania Sud’ (protocol no. 124_r.p.s.o. – May 31, 2023). The Declaration of Helsinki was applied, and participation was anonymous and voluntary [42]. Informed consent was obtained electronically before the survey.

## 3 Results

Data collection and management were conducted in REDCap; incomplete responses were excluded. Descriptive analyses (frequencies, percentages) are reported in Table 1. The final sample included 212 nurses (63.2% women) with ages 23–57 years (*M* = 38.57, SD = 8.78). Overall, 44.8% reported a postgraduate specialisation, 32.6% of whom in Critical Care; additionally, 15.1% held a master's degree. By department, 25.0% worked in Surgery/Operating Room, 13.2% in Medical, 39.6% in Emergency/Intensive Care and 22.2% in Territorial Emergency Services (i.e., hospital‐affiliated prehospital emergency services/EMS units).

**TABLE 1:** Sociodemographic characteristics.

Variable	*n* (%) or mean (SD)
Gender	
Female	134 (63.2)
Age (years)	38.57 (8.78)
Years of experience	
0–9	59 (27.8)
10–20	77 (36.3)
> 20	76 (35.8)
Citizenship	
Foreign	8 (3.8)
Postgraduate specialisation	
Yes	95 (44.8)
Postgraduate type (*n* = 86)	
Critical area	28 (32.6)
Coordination	36 (41.9)
Operating room	13 (15.1)
Forensic	1 (1.2)
Wound care	2 (2.3)
Community nurse	1 (1.2)
Vascular access	5 (5.8)
Master degree	
Yes	32 (15.1)
Work area	
North	52 (24.5)
Centre	109 (51.4)
South	51 (24.1)
Department	
Surgery unit/Operating room	53 (25.0)
Medicine	28 (13.2)
Emergency room	26 (12.3)
Resuscitation	27 (12.7)
Intensive room	31 (14.6)
Territorial emergency	47 (22.2)

*Note:* Percentages may not total 100 due to rounding.

### 3.1 Dimensionality of the FPDIP/R‐NPPQ

No missing data were identified among the 37 items of the FPDIP/R‐NPPQ, all of which were framed as positive statements rated on a 5‐point Likert questionnaire (Table 2).

**TABLE 2:** Descriptive statistics.

	*N*	Mean	SD	Skewness	SE of skewness	Kurtosis	SE of kurtosis
	Missing						
N1. I believe that family is a fundamental presence that should be involved at all times in a person's life	0	2.92	0.687	−1.134	0.167	2.215	0.333
N2. I believe that the presence of family members would not cause delays in critical interventions or prolonged resuscitation efforts	0	1.72	1.116	0.330	0.167	−1.245	0.333
N3. I believe that the presence of family members can influence my performance during invasive procedures or CPR	0	1.25	0.938	0.802	0.167	−0.236	0.333
N4. I believe that family presence during invasive procedures or CPR is feasible, provided it does not interfere with the emergency team's activities	0	2.39	0.984	−0.822	0.167	−0.797	0.333
N5. I feel adequately prepared to manage the presence of family members during invasive procedures or CPR	0	1.68	1.026	0.384	0.167	−1.302	0.333
N6. I believe that the absence of family members during invasive procedures or CPR is beneficial for nurses/emergency teams	0	1.37	0.807	0.327	0.167	−0.305	0.333
N7. I believe that witnessing invasive procedures or CPR can lead to post‐traumatic stress for family members	0	0.96	0.595	1.240	0.167	4.421	0.333
N8. I believe that the presence of family members during invasive procedures or CPR increases the stress levels of nurses	0	0.94	0.675	1.099	0.167	2.684	0.333
N9. I believe that family presence can help improve the quality of care provided to the patient	0	1.93	0.995	0.075	0.167	−1.650	0.333
N10. I believe that family members would want to be present during critical procedures	0	2.42	0.938	−0.443	0.167	−0.595	0.333
N11. I believe that allowing family presence during invasive procedures helps them witness the resuscitation team's efforts and the completeness of care, potentially reducing the risk of complaints or disputes	0	2.31	0.981	−0.526	0.167	−1.180	0.333
N12. I believe that family presence facilitates the grieving process when resuscitation is unsuccessful	0	2.05	1.029	−0.042	0.167	−1.554	0.333
N13. I believe that family presence provides emotional support to the patient during invasive procedures or CPR	0	1.76	0.990	0.410	0.167	−1.344	0.333
N14. I believe that family presence encourages emotional and physical contact between the patient and the family member, providing greater comfort for both	0	1.78	0.998	0.476	0.167	−1.065	0.333
N15. I believe that family presence can reduce fear, distress and anxiety in family members	0	1.57	0.934	0.616	0.167	−0.591	0.333
N16. I believe that family presence allows quicker access to information about the patient's clinical condition and progression	0	2.83	0.883	−0.901	0.167	0.438	0.333
N17. I believe that family members who are excluded from emergency care often feel dissatisfied due to a lack of information and support	0	2.93	0.776	−1.236	0.167	2.076	0.333
N18. I believe that family presence can create space limitations that hinder proper clinical functioning	0	1.13	0.813	1.096	0.167	1.043	0.333
N19. I feel supported by my colleagues when family members are present during procedures	0	1.27	0.938	0.934	0.167	0.471	0.333
F1. I would support the implementation of family presence practices through staff training, including the development of policies and protocols	0	1.27	0.938	0.934	0.167	0.471	0.333
F2. I believe that staff training has little impact on attitudes towards family presence during invasive procedures or CPR	0	2.40	0.961	−0.523	0.167	−0.785	0.333
F3. I believe that resuscitation and invasive procedure simulations—where staff members role‐play as patients and family members—are useful training tools to prepare emergency teams for family presence	0	2.40	0.951	−0.613	0.167	−0.921	0.333
F4. I feel comfortable communicating with family members about what is happening during invasive procedures or CPR	0	2.77	0.859	−0.804	0.167	0.344	0.333
F5. I believe that assessing a patient's wish to have a family member present during invasive procedures, as part of medical history‐taking, could be useful	0	2.69	0.852	−0.945	0.167	0.358	0.333
F6. I believe it is helpful, during hospitalisation, to observe the behaviour of family members to identify those who might interfere with the emergency team during invasive procedures	0	2.67	0.967	−0.587	0.167	−0.648	0.333
F7. I believe that specific policies are needed within care units regarding family presence during invasive procedures and/or CPR	0	3.45	0.774	−1.727	0.167	3.424	0.333
F8. For families who will be present, I believe it is useful to prepare them in advance regarding the patient's condition, the procedures being performed, required protective equipment, and what they might see, hear or smell	0	2.97	0.715	−1.294	0.167	3.012	0.333
F9. I believe that video calls during invasive procedures may be a useful way to involve family members, even though they do not allow for physical contact	0	3.18	0.567	−1.096	0.167	6.381	0.333
O1. I believe that having a dedicated staff member to prepare and support the family during invasive procedures or CPR is necessary	0	1.02	0.851	0.932	0.167	1.054	0.333
O2. I believe that a dedicated staff member can improve family integration during invasive procedures or CPR	0	3.11	0.636	−0.768	0.167	2.094	0.333
O3. I believe that a dedicated staff member can help reduce my workload during invasive procedures or CPR	0	3.02	0.688	−0.993	0.167	2.107	0.333
O4. I believe that a dedicated staff member can provide effective emotional support to family members during invasive procedures or CPR	0	3.02	0.757	−0.834	0.167	0.994	0.333
O5. I believe that a dedicated staff member can improve communication between the healthcare team and family members	0	2.84	1.149	−1.631	0.167	1.840	0.333
O6. I believe it is helpful to have a staff member who prepares family members emotionally before invasive procedures or CPR begin	0	2.90	0.656	−1.519	0.167	3.366	0.333
O7. I believe that specific training for staff dedicated to managing family presence during invasive procedures or CPR is essential	0	3.12	0.560	−0.457	0.167	2.297	0.333
O8. I believe that the presence of a dedicated staff member improves the overall emotional well‐being of family members during invasive procedures or CPR	0	3.15	0.655	−0.875	0.167	2.191	0.333
O9. I believe that my healthcare institution should invest more in the training and hiring of dedicated staff for managing family presence during invasive procedures or CPR	0	3.58	0.615	−1.402	0.167	2.040	0.333

The results of parallel analysis, reported in Table 3, indicated retaining three factors. Subsequently, a three‐factor EFA was performed using principal axis factoring and Oblimin oblique rotation. The resulting solution represented 41.68% of the total variance. The factor correlations ranged from |0.47| to |0.52|, indicating moderate to strong associations among factors and justifying the choice of an oblique rotation.

**TABLE 3:** The first 10 factors extracted by the EFA.

Component	Initial eigenvalues			
	Total	% of Variance	Cumulative %	95th percentile of the simulated eigenvalues
1	10.582	28.601	28.601	2.002
2	2.937	7.938	36.539	1.886
3	1.902	5.142	41.680	1.780
4	1.656	4.475	46.156	1.692
5	1.470	3.973	50.129	1.618
6	1.275	3.445	53.574	1.557
7	1.197	3.236	56.809	1.502
8	1.148	3.101	59.911	1.442
9	1.098	2.967	62.878	1.384
10	0.981	2.652	65.530	1.339

*Note:* Empirical eigenvalues and percentages of explained variance are reported along with the 95th percentile of the simulated eigenvalues.

Abbreviation: EFA, exploratory factor analysis.

Item loadings were further assessed using the marker index [47], a rigorous method comparing primary and secondary loadings to minimise cross‐loadings and optimise factorial clarity. A total of 19 elements surpassed the recommended marker index threshold of 0.40 [46] and were therefore retained as valid indicators of their respective factors in the final version of FPDIP/R‐NPPQ. The results confirmed that each item demonstrated a clear primary load on a single factor, with secondary loadings near zero on the remaining factors. A subsequent three‐factor EFA performed on the selected items accounted for 57.47% of the total variance, with factor correlations ranging from |0.40| to |0.58|. The identified factors are described in Table 4 in order of the explained variance. Factor 1 was defined by five items that assess the extent to which healthcare professionals attribute a positive value to the presence of family members for the well‐being of both patients and relatives, encompassing aspects of comfort, anxiety reduction and perceived emotional benefit. Consequently, it was labelled ‘Emotional value and relational benefits of family presence’. Factor 2 comprised six items and reflects the perception of being able (or the need) to prepare, inform and integrate family members into critical situations through effective communication, policies and procedures and targeted training. Therefore, it was named ‘Communication skills and structured management of family presence’. Factor 3 consisted of eight items that indicate the perceived importance and usefulness of having a professional specifically assigned to supporting family members, providing benefits for both the clinical team and the relatives. Consequently, it was designated ‘Operational support through a dedicated staff member’.

**TABLE 4:** Three‐factor solution of the EFA in order of explained variance.

		F1	F2	F3
N2	I believe that the presence of family members would not cause delays in critical interventions or prolonged resuscitation efforts.	**0.410**	0.080	0.064
N13	I believe that family presence provides emotional support to the patient during invasive procedures or CPR.	**0.907**	−0.016	0.005
N14	I believe that family presence encourages emotional and physical contact between the patient and the family member, providing greater comfort for both.	**0.935**	0.007	0.001
N15	I believe that family presence can reduce fear, distress and anxiety in family members.	**0.812**	0.002	−0.036
F1	I would support the implementation of family presence practices through staff training, including the development of policies and protocols.	**0.465**	0.022	0.063
F2	I believe that staff training has little impact on attitudes towards family presence during invasive procedures or CPR.	0.161	**0.486**	0.253
F3	I believe that resuscitation and invasive procedure simulations—where staff members role‐play as patients and family members—are useful training tools to prepare emergency teams for family presence.	0.119	**0.431**	0.171
F4	I feel comfortable communicating with family members about what is happening during invasive procedures or CPR.	−0.021	**0.924**	−0.061
F5	I believe that assessing a patient's wish to have a family member present during invasive procedures, as part of medical history‐taking, could be useful.	0.013	**0.686**	0.097
F6	I believe that it is helpful, during hospitalisation, to observe the behaviour of family members to identify those who might interfere with the emergency team during invasive procedures.	0.102	**0.616**	−0.030
F8	For families who will be present, I believe it is useful to prepare them in advance regarding the patient's condition, the procedures being performed, required protective equipment, and what they might see, hear or smell.	−0.059	**0.545**	0.083
O2	I believe that a dedicated staff member can improve family integration during invasive procedures or CPR.	0.030	0.108	**0.582**
O3	I believe that a dedicated staff member can help reduce my workload during invasive procedures or CPR.	−0.007	0.151	**0.665**
O4	I believe that a dedicated staff member can provide effective emotional support to family members during invasive procedures or CPR.	−0.023	−0.001	**0.735**
O5	I believe that a dedicated staff member can improve communication between the healthcare team and family members.	0.043	−0.018	**0.697**
O6	I believe that it is helpful to have a staff member who prepares family members emotionally before invasive procedures or CPR begin.	0.025	−0.056	**0.771**
O7	I believe that specific training for staff dedicated to managing family presence during invasive procedures or CPR is essential.	0	0.087	**0.637**
O8	I believe that the presence of a dedicated staff member improves the overall emotional well‐being of family members during invasive procedures or CPR.	−0.045	−0.074	**0.713**
O9	I believe that my healthcare institution should invest more in the training and hiring of dedicated staff for managing family presence during invasive procedures or CPR.	0.035	−0.076	**0.499**

*Note:* The values in bold indicate the item’s saturation on that factor. Therefore, any value greater than or equal to 0.40 indicates good saturation on the main factor.

Cronbach's *α* was calculated for each latent factor to assess internal consistency. The highest Cronbach's *α* was observed for Factor 3 (*α* = 0.87), followed by Factor 1 (*α* = 0.82) and Factor 2 (*α* = 0.82).

### 3.2 Confirmatory Factor Analyses on the FPDIP/R‐NPPQ

**FIGURE 1:**
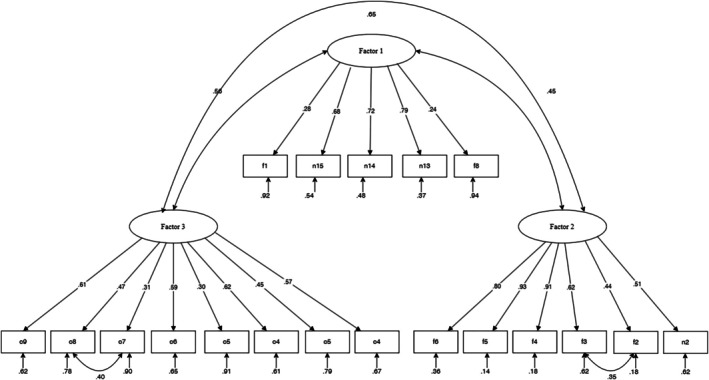
CFA conducted on FPDIP/R‐NPPQ. The figure illustrates factorial relationship emerging from CFA.

CFA was conducted on FPDIP/R‐NPPQ to test the factor structure that emerged from EFA (Figure 1). Given that some items of the FPDIP/R‐NPPQ showed an asymmetry higher than |1.0|, maximum likelihood (i.e., MLR) was used as robust estimator in the following analyses to avoid problems related to data non‐normality. The Mardia test confirmed the need to use a robust estimator (multivariate skewness: *M* = 37.16, SD = 1.51, *p* < 0.001; multivariate kurtosis: *M* = 395.39, SD = 3.29, *p* < 0.001). In the present study, the null model for FPDIP/R‐NPPQ produced an RMSEA of 0.210. This result indicates that incremental fit indices must be considered to evaluate model fit.

Starting from an initial factorial model, the CFA showed unacceptable fit indices for CFI = 0.85, TLI = 0.82, and SRMR = 0.09, with the saturation of item F8 resulted not significant. From the analysis of the modification indices, we considered two items with cross‐loading, item N2 and item F8. Specific residual covariances were added for these item pairs: O7 and O8, F2 and F3. These modifications addressed methodological artefacts consistent with the proximity effect described by Weijters et al. [51], whereby adjacent positively worded items demonstrate correlated error terms. The CFA on the model thus conducted showed acceptable fit indices (RMSEA = 0.05, 90% CI = 0.04–0.06, CFit = 0.36, CFI = 0.93, TLI = 0.92, SRMR = 0.07), which confirms a good factor structure. The standardised item loadings ranged from |0.24| to |0.92|, all significant. Factor reliability was estimated using McDonald's *ω* [53], Omega to overcome Cronbach's *α* limitations, specifically the violation of tau equivalence [52]. Omega ranged from 0.82 to 0.87, indicating good to excellent internal consistency of the factors. The correlation coefficients were significant and positive, revealing weak to moderate associations among the FPDIP/R‐NPPQ dimensions. The highest correlation coefficient was detected between Factor 2 and Factor 3 (*r* = 0.70), followed by that between Factor 1 and Factor 3 (*r* = 0.56), and finally by that between Factor 1 and Factor 2 (*r* = 0.45).

## 4 Discussion

This study developed and validated a measure of Italian nurses' perceptions and preparedness for FPDIP/R using a two‐step psychometric strategy in a multicentre sample (*n* = 212), following best practices for ordinal data and model evaluation [54, 55]. Existing questionnaires have examined risk–benefit and self‐confidence in FPDIP/R [21, 56], but evidence is lacking on healthcare professionals' preparedness for and perceptions of FPDR as distinct, measurable constructs.

The questionnaire was initially composed of 52 items; following the removal of dichotomous and open‐ended questions, psychometric analyses were conducted on 37 five‐point Likert items. A pilot study with the first 30 respondents identified 18 items as redundant, a finding that was consistent with their low factor loadings (< 0.40) reported in Table 5. The final instrument comprises 19 items with factor loadings > 0.40 in EFA.

**TABLE 5:** Eliminated items.

Code	Item
F7	I believe that specific policies are needed within care units regarding family presence during invasive procedures and/or CPR
F9	I believe that video calls during invasive procedures may be a useful way to involve family members, even though they do not allow for physical contact
N1	I believe that family is a fundamental presence that should be involved at all times in a person's life
N10	I believe that family members would want to be present during critical procedures
N11	I believe that allowing family presence during invasive procedures helps them witness the resuscitation team's efforts and the completeness of care, potentially reducing the risk of complaints or disputes
N12	I believe that family presence facilitates the grieving process when resuscitation is unsuccessful
N16	I believe that family presence allows quicker access to information about the patient's clinical condition and progression
N17	I believe that family members who are excluded from emergency care often feel dissatisfied due to a lack of information and support
N18	I believe that family presence can create space limitations that hinder proper clinical functioning
N19	I feel supported by my colleagues when family members are present during procedures
N3	I believe that the presence of family members can influence my performance during invasive procedures or CPR
N4	I believe that family presence during invasive procedures or CPR is feasible, provided it does not interfere with the emergency team's activities
N5	I feel adequately prepared to manage the presence of family members during invasive procedures or CPR
N6	I believe that the absence of family members during invasive procedures or CPR is beneficial for nurses/emergency teams
N7	I believe that witnessing invasive procedures or CPR can lead to post‐traumatic stress for family members
N8	I believe that the presence of family members during invasive procedures or CPR increases the stress levels of nurses
N9	I believe that family presence can help improve the quality of care provided to the patient
O1	I believe that having a dedicated staff member to prepare and support the family during invasive procedures or CPR is necessary

*Note:* Items removed due to marker‐index, cross‐loading criteria (< 0.40), comments and indications of the first 30 responder testers.

Factor 1 included five items and was labelled emotional value and relational benefits of family presence. This factor reflects the psycho‐emotional dimension that often motivates family presence during invasive procedures, including potential mitigation of post‐traumatic sequelae when resuscitation has an adverse outcome and facilitation of healthier bereavement processing [9, 57]. It also encompasses awareness of the procedure and reduced hostility or aggression towards staff through transparent engagement [58, 59]. In nursing, this domain aligns with the profession's ethical–moral commitments—prioritising psychological support for relatives and acknowledging the need for a family member's presence during procedures that profoundly challenge human sensitivity, such as resuscitation and other invasive interventions [60, 61]. Factor 1 is congruent with the construct articulated by Twibell et al.—the ‘benefits/value’ dimension of risk–benefit scales—where risk and benefit are filtered through emotional appraisal [56].

Factor 2 comprised six items and was named communication skills and structured management of family presence. For nurses, the capacity to communicate the critical event, describe ongoing actions and manage the situation is fundamental to FCC [62]. Comprehensive, explanatory communication—either describing what is being done or preparing relatives for what will occur—is essential to ready family members for a novel and emotionally charged situation [63]. Communication functions as a negotiation, particularly during high‐intensity events such as CPR, where staff must both provide information and receive feedback to gauge the relative's comprehension and lucidity; moreover, as reflected in the questionnaire, relatives can be key informants for clinical history and patient‐specific information, this communication method reflects the person‐centred care and communication continuum (PC4) model [63]. This factor also substantiates the preparedness domain in FPDIP/R—overlapping with self‐confidence/preparedness (communication, policy, simulation) reported in self‐confidence scales and training‐impact studies [21, 56, 64].

Factor 3 consisted of eight items and was designated operational support through a dedicated staff member. This factor addresses cultural barriers to FPDIP/R by indicating that, for nurses, family presence is desirable but should occur only when supported by an experienced team with strong communication skills [65]. Having a team that specifically attends to relatives is seen as essential to implementing family‐centred care [66]. In practice, the FPDIP/R team acts as a buffer: it enables clinicians to focus on the patient while concurrently ‘caring for’ the relative's psycho‐physical and social needs during the procedure [67]. Training is viewed as mandatory; ethically sensitive practices can be implemented when experts and clear procedures govern the presence of a lay relative during invasive clinical interventions [68]. Recent studies suggest that witnessing resuscitation without a dedicated support figure may create a dual risk: unmitigated interference by relatives with potential adverse procedural outcomes [61], and psychological trauma for the relative who is unable to process the graphic nature of the event, with possible progression to anxiety and depression [69]. This factor makes explicit the organisational domain—often discussed as an ‘enabler’ or the ‘family facilitator’ in surveys and reviews [70, 71];—but rarely modelled as an autonomous latent dimension. Here, it is operationalised as a coherent construct with eight indicators (O2–O9). The facilitator role is pivotal, ensuring safety, sterility and appropriate prioritisation for relatives and fostering equipoise and consensus within the clinical team during the procedure [72].

Overall, the psychometric architecture is robust, as CFA confirms a parsimonious and theoretically grounded three‐factor solution. The instrument extends previous FPDR measures by introducing an explicit organisational factor—operational support through a dedicated staff member—which is critical for the safe, ethical and effective implementation of family presence during invasive procedures and resuscitation. While the emotional and relational value of family presence and the importance of communication skills are well recognised in the FPDR literature, the identification of operational support through a dedicated staff member as an independent latent dimension represents a substantial advancement. This factor operationalises what has often been described narratively—as the ‘family facilitator’ or ‘enabler’—into a measurable organisational construct.

From a managerial perspective, the FPDIP/R‐NPPQ functions as more than an attitudinal scale. It provides nurse managers, clinical leaders and policymakers with a diagnostic tool to assess baseline staff readiness before implementing FPDR/FPDIP policies; identify specific gaps in training, communication processes or organisational support; allocate human resources (e.g., identifying the need for a trained family facilitator); and monitor the impact of educational or policy interventions over time.

In invasive and resuscitative procedures, where time pressure, emotional intensity and risk converge, the absence of structured organisational support can increase procedural interference, staff stress and family psychological harm. By explicitly measuring organisational preparedness, the FPDIP/R‐NPPQ supports safer implementation of family presence, reducing variability in practice and enabling clinicians to focus on technical performance while ensuring appropriate support for relatives.

### 4.1 Implications for Practice and Further Research

**FIGURE 2:**
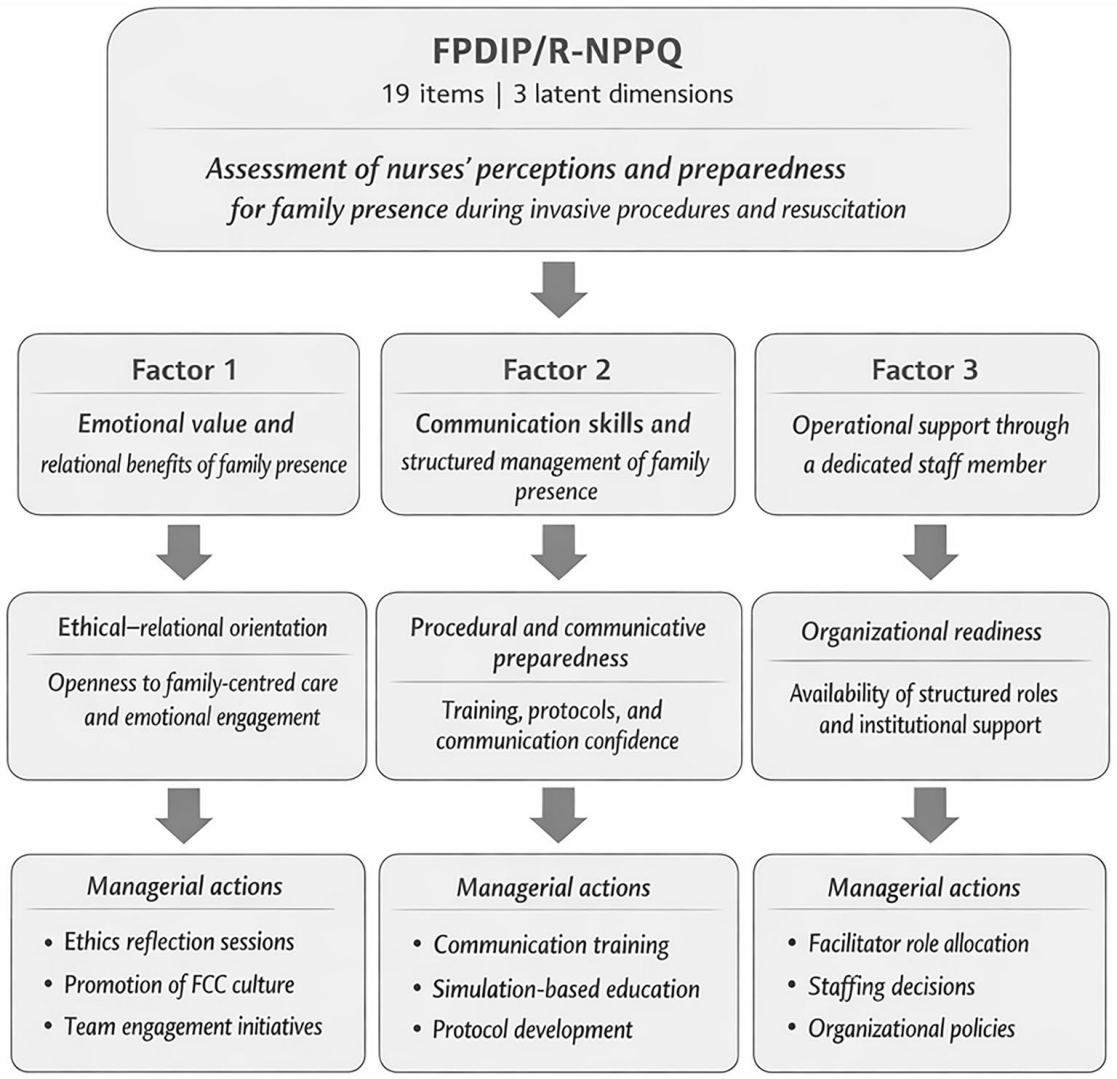
Conceptual framework for interpreting FPDIP/R‐NPPQ subscale scores and their managerial implications. The figure illustrates the structure of the FPDIP/R‐NPPQ and the interpretive logic of its three latent dimensions: emotional value and relational benefits of family presence, communication skills and structured management of family presence and operational support through a dedicated staff member. Subscale scores are intended to be interpreted at the dimension level rather than as a single total score or against predefined cut‐off values. Each dimension provides complementary information regarding attitudinal orientation, procedural preparedness and organisational readiness for family presence during invasive procedures and resuscitation. The framework highlights how subscale profiles can inform targeted educational, organisational and policy‐level interventions, without implying diagnostic care implementation.

The FPDIP/R‐NPPQ can be integrated into routine clinical governance as a decision‐support and quality‐improvement instrument, supporting managers and clinical leaders in translating the abstract concept of preparedness for family presence into actionable domains. As illustrated in Figure 2, the questionnaire provides an interpretive framework linking nurses' perceptions and preparedness across three latent dimensions—emotional–relational orientation, procedural and communicative preparedness and organisational readiness—to targeted managerial actions. Moving beyond a sole focus on individual attitudes, this framework emphasises integrated organisational preparedness, enabling healthcare systems to convert ethical intentions into safe, structured and sustainable practice.

From a clinical and managerial perspective, the instrument functions as a bridge between policy and bedside care by facilitating the anticipation of risks prior to FPDR/FPDIP implementation, supporting the structured allocation of the family facilitator role, reducing nurses' emotional burden during invasive and resuscitative procedures and improving alignment within the patient–family–team triad in highly stressful situations. By embedding the FPDIP/R‐NPPQ within education, audit and implementation cycles, healthcare organisations can systematically enhance the quality, safety and equity of invasive and resuscitative care while promoting adherence to family‐centred care principles.

More broadly, the questionnaire enriches the FPDIP/R framework by offering a concrete tool to assess educational appropriateness and organisational readiness, providing an innovative window into nurses' perceptions during invasive procedures that have traditionally been conducted at a distance from relatives. Future research should examine measurement invariance across settings and professions, establish test–retest reliability and responsiveness and explore associations with behavioural, patient‐ and family‐centred outcomes, as well as key workforce indicators such as burnout, intention to leave and job satisfaction among nurses.

### 4.2 Limitations and Strengths

The first limitations, reducing items from 37 to 19, enhanced factorial clarity but may have narrowed content coverage. Second, two residual covariances were freed to address proximity/method effects; while theoretically justified, the resulting correlated errors reduce parsimony and may reflect sample‐specific patterns. Third, validation was conducted only among Italian nurses; cross‐cultural and cross‐professional validation is required before broader dissemination.

The first strengths, multicentre sampling across ICUs, EDs, operating theatres and hospital‐affiliated prehospital emergency services captures real‐world organisational variability, enhancing external validity. Second, the final 19‐item, three‐factor model demonstrated good global fit and reliability, and its brevity facilitates clinical implementation without sacrificing measurement quality. Third, the explicit operational support through a dedicated staff member factor identifies a rarely measured implementation determinant, extending beyond traditional risk–benefit and self‐confidence frameworks and supporting translation into practice. This third strength is highly innovative compared with other questionnaires available in the literature and aligns perfectly with the emerging clinical needs of nurses working in these critical settings.

## 5 Conclusions

The FPDIP/R‐NPPQ is a psychometrically sound, theory‐grounded instrument for assessing nurses' perceptions and preparedness for family presence during invasive procedures and resuscitation. The three‐factor structure demonstrated good global fit and satisfactory internal consistency, while model refinements addressed proximity effects without permitting cross‐loadings, thereby preserving construct distinctiveness and alignment with existing FPDR measures. Importantly, the instrument makes explicit the organisational ‘family facilitator’ dimension, which is often implicitly acknowledged but rarely measured.

These findings indicate that the FPDIP/R‐NPPQ can serve multiple purposes within resuscitation systems of care, including baseline assessment of staff readiness, identification of training needs related to communication and family integration and evaluation of organisational policies and implementation strategies consistent with family‐centred care principles. By operationalising organisational readiness alongside attitudinal and procedural dimensions, the questionnaire provides managers and clinical leaders with a practical tool to support structured, safe and sustainable implementation of family presence during invasive and resuscitative procedures.

## Author Contributions

**Davide Bartoli:** writing – review and editing (equal), software (lead), data curation (lead), conceptualization (lead), writing – review and editing (equal), validation (lead), supervision (lead). **Francesca Trotta:** investigation (lead), writing – original draft preparation (lead), writing – review and editing (lead), supervision (equal). **Mariachiara Figura:** writing – review and editing (equal), supervision (equal). **Marco Di Muzio:** review and editing. **Daniele Napolitano:** writing – review and editing (supporting), supervision (equal). **Marco Fatata:** investigation (lead), data curation (equal), formal analysis (supporting). **Gianluca Pucciarelli:** validation (lead), supervision (equal). **Francesco Petrosino:** writing – review and editing (equal), software (lead), data curation (lead), formal analysis (lead), supervision (equal).

## Funding

This study was funded by the Center of Excellence for Nursing Scholarship (CECRI), Rome, Italy, with reference number 3900 1/04/2025.

## Consent

Informed consent was obtained from the nurses involved. No patients were involved.

## Conflicts of Interest

The authors declare no conflicts of interest.

## Supporting information


**Appendix S1:** Guidelines for Reporting Reliability and Agreement Studies (GRRAS).

## Data Availability

The data that support the findings of this study are available from the corresponding author upon reasonable request.
