# Supplementary material for: Family Presence During Invasive Procedures or Resuscitation, Nurse Perception and Preparedness Questionnaire (FPDIP/R‐NPPQ): A Validity and Reliability Study
Source: Nurs Crit Care. 2026 Apr 6;31(3):e70470. doi: 10.1111/nicc.70470 (PMC13051457; doi:10.1111/nicc.70470)
Supplement: Supplementary file 1 — Appendix S1: Guidelines for Reporting Reliability and Agreement Studies (GRRAS). [file NICC-31-0-s001.docx]

**Appendix S1.** Guidelines for Reporting Reliability and Agreement Studies (GRRAS).

| SECTION ITEM RESPONSE | | |
| --- | --- | --- |
| TITLE AND  ABSTRACT | 1. Identify in title or abstract that interrater/intrarater reliability or agreement was investigated. | YES |
|  | 1. Name and describe the diagnostic or measurement device of interest explicitly. | YES |
|  | 1. Specify the subject population of interest | YES |
|  | 1. Specify the rater population of interest (if applicable) | YES |
|  | 1. Describe what is already known about reliability and agreement and provide a rationale for the study (if applicable). | YES |
| METHODS | 1. Explain how the sample size was chosen. State the determined number of raters, subjects/objects, and replicate observations. | YES |
|  | 1. Describing the sampling method | YES |
|  | 1. Describing the measurement/rating process (e.g. interval between repeated measurements, availability of clinical information, blinding). | YES |
|  | 1. State whether measurements/rating were conducted independently | YES |
|  | 1. Describe the statistical analysis | YES |
| RESULTS | 1. State the actual number of raters and subjects/objects which were included and the number of replicate observations which were conducted. | YES |
|  | 1. Describe the sample characteristics of raters and subjects (e.g. training, experience). | YES |
|  | 1. Report estimates of reliability and agreement including measures of statistical uncertainty. | YES |
| DISCUSSION | 1. Discuss the practical relevance of results. | YES |
| AUXILIARY  MATERIAL | 1. Provide detailed results if possible (e.g. online) | Only upon request by the main author |

*Version based on Table I in: Kottner J, Audigé L, Brorson S, Donner A, Gajeweski BJ,*

*Hróbjartsson A, Robersts C, Shoukri M, Streiner DL. Guidelines for reporting reliability and*

*agreement studies (GRRAS) were proposed. J Clin Epidemiol. 2011;64(1):96-106*
